# Generation of the Human Biped Stance by a Neural Controller Able to Compensate Neurological Time Delay

**DOI:** 10.1371/journal.pone.0163212

**Published:** 2016-09-21

**Authors:** Ping Jiang, Ryosuke Chiba, Kaoru Takakusaki, Jun Ota

**Affiliations:** 1 Department of Precision Engineering, School of Engineering, The University of Tokyo, Tokyo, Japan; 2 Research Center for Brain Function and Medical Engineering, Asahikawa Medical University, Asahikawa, Japan; 3 Research into Artifacts, Center for Engineering (RACE), The University of Tokyo, Kashiwa, Japan; Ludwig-Maximilians-Universitat Munchen, GERMANY

## Abstract

The development of a physiologically plausible computational model of a neural controller that can realize a human-like biped stance is important for a large number of potential applications, such as assisting device development and designing robotic control systems. In this paper, we develop a computational model of a neural controller that can maintain a musculoskeletal model in a standing position, while incorporating a 120-ms neurological time delay. Unlike previous studies that have used an inverted pendulum model, a musculoskeletal model with seven joints and 70 muscular-tendon actuators is adopted to represent the human anatomy. Our proposed neural controller is composed of both feed-forward and feedback controls. The feed-forward control corresponds to the constant activation input necessary for the musculoskeletal model to maintain a standing posture. This compensates for gravity and regulates stiffness. The developed neural controller model can replicate two salient features of the human biped stance: (1) physiologically plausible muscle activations for quiet standing; and (2) selection of a low active stiffness for low energy consumption.

## Introduction

Stance postural control (SPC), which allows individuals to maintain an upright stance, is one of the most important and basic requirements for a comfortable life [1]. The physiologically inspired neural controller (NC) model for SPC has potential applications in a variety of fields. For example, the NC model could be used to assist device development and design robotic control systems. To realize such applications, the model must successfully replicate the functionality of the human neural controller (e.g., the ability to maintain a posture via muscle coordination, while compensating for the neurological time delay). The realized model must also replicate the salient features (e.g., physiologically plausible muscle activations) of the human biped stance in computer simulations.

System identification is one approach to developing an NC model based on experimentally measured data [2–13]. Applications of this method aim to develop an NC model that can simulate human movement that agrees with experimental data. To achieve this, the variables in the NC model are typically identified by minimizing the discrepancies between the simulation results and the corresponding experimental data. Using system identification, researchers have studied the influence on SPC of joint and muscle stiffness [3–6], sensory information [7–10, 12, 13], feedback (fb) gains [14–17], and the muscle torque generation process [11]. Several of the studies cited above [2–19] have proposed potential NC models, such as the stiffness control [3], sensory fb control [17], and disturbance estimation and compensation (DEC) [20] models. An optimal postural control model was proposed by Qu et al. [21] to identify the change of balance control mechanism due to aging. This model was further adopted to investigate the role of passive and active toque for maintaining postural balance [22]. A sliding model [23] developed by Zhang et al., enables the passive and active joint stiffness to be estimated. He also proposed a novel identification approach to estimate passive and active joint stiffness without any additional perturbations [24]. However, the efficacy of these models is solely determined by their ability to reproduce experimental data, and it is difficult to confirm whether they truly reflect the mechanism behind human postural control. In addition, it is unclear whether these models can be employed to control a more complex human body model (e.g., a musculoskeletal model with many muscles).

Forward modeling is another effective approach for developing NC models. Different from system identification, forward modeling does not use any experimental data as input. Instead, the variables in the NC model are typically designed by optimizing an assumed performance criterion (e.g., the joint angle displacement) [25]. It is possible to validate an NC model developed using forward modeling by examining whether the simulated results reflect the salient features of human movement, and comparing those results with experimental data. To date, three types of NC model that generate the biped stance have been proposed. The first is based on conventional feed-forward (ff) control in conjunction with fb control [26–30], whereby the neural controller estimates the state of the human body internal model [31] (the joint angle and angular velocity) using an optimal estimator [32]. Torque is added directly using a proportional-differential (PD) controller that utilizes the estimated joint kinematics information [26]. For this model, van der Kooij et al. [26] have reported that the controller can compensate for a delay of approximately 80 ms, and that it can simulate the salient features of sensation integration observed in experiments. The second model type is based on fb control only [18, 33–35]. For an inverted pendulum model and assuming fb control only, Masani et al. [18] reported that a PD controller can compensate for a delay of approximately 185 ms, provided the gain is sufficiently high. The designed model can reproduce the position and velocity of the center of mass (CoM), along with the joint torque data. The third model type employs intermittent control [36–42], whereby the PD fb controller is activated intermittently based on the status of the joint angle.

To generate a human-like biped stance that reflects the appropriate salient features, an NC model should: (1) successfully maintain a musculoskeletal model with muscles in a standing posture; and (2) compensate for the neurological time delay. Many previous studies [4, 5, 7–19, 26, 28–30, 33–38, 40–44] have utilized simplified musculoskeletal models with fewer joints and torque actuators in place of muscle actuators. They have also neglected the human musculoskeletal anatomy (e.g., the CoM of each body segment, muscle attachment points, and skeletal inertial properties). These previous studies have employed one [4, 5, 7–9, 11, 13–16, 18, 30, 36–38], two [10, 12, 17, 19, 29, 40–42, 44], or three degrees of freedom (DoF) [26, 28, 43] inverted pendulum models with no muscles, a one-DoF inverted pendulum model with one [33] or three muscles [34], or a three-DoF inverted pendulum model with nine muscles [35]. However, Günther et al. [43] and Hsu et al. [45] have suggested that all the joints should contribute to the biped stance. Further, Horlings et al. [46] identified the importance of muscle strength to postural stability. Therefore, a musculoskeletal model that more accurately represents the human anatomy than the above inverted pendulum models should be developed. For example, Clark et al. [47] have used a reflex controller to hold a musculoskeletal model with 92 muscles in a standing position; however, neurological time delays similar to those of humans was not considered, and simulated muscle activations were not evaluated. Further, a DEC model [10, 20, 48] has been successfully implemented in a robot featuring eight muscles, incorporating a realistic time delay. However, a robot with more muscles and more physiologically accurate anatomy is needed to approximate the human anatomy. To date, no NC model that can compensate for the neurological time delay in the case of a more human-like, complex musculoskeletal model in a standing posture has been developed.

In this study, we employ a forward modeling method to develop a computational model of a neural controller that can simulate a human-like biped stance and yield physiologically plausible features for this stance.

**Fig 1:**
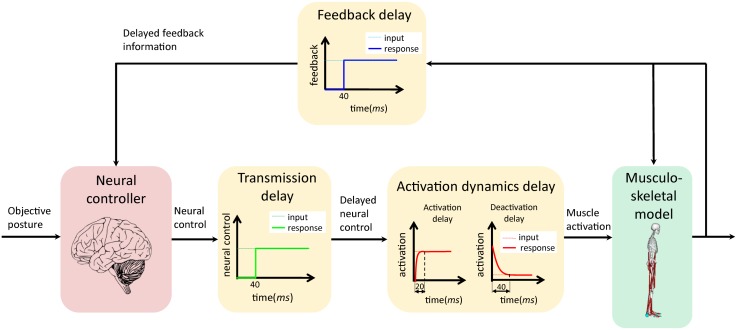
Human stance postural control. Red block: neural controller to actuate muscles during quiet standing. Green block: musculoskeletal model. Flesh-colored blocks: 120-ms neurological time delay. The delay is comprised of a 40-ms feedback delay, 40-ms transmission delay, and (at most) 40-ms activation dynamics delay. The activation dynamics delay corresponds to a 20-ms activation time delay during the muscle activation process and a 40-ms deactivation time delay during the muscle deactivation process.

Specifically, we aim to develop an NC model (red block in Fig 1) capable of: (1) realizing human SPC (Fig 1); and (2) reflecting the features that generate muscle activations within physiologically plausible ranges for the human biped stance. In realizing this postural control, we derive a musculoskeletal model (green block in Fig 1) with 70 muscles and seven joints in a standing position under the influence of a 120-ms neurological time delay (flesh-colored blocks in Fig 1).

## Methods

**Fig 2:**
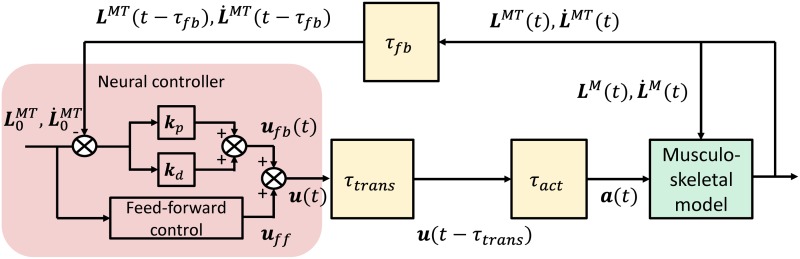
Proposed NC model for human stance postural control. ***u***, ***u***_*ff*_, and ***u***_*fb*_ are the total, ff, and fb controls, respectively; *τ*_*fb*_, *τ*_*trans*_, and *τ*_*act*_ are the feedback, transmission, and activation dynamics delay, respectively. ***L***^*MT*^ and ${\dot{L}}^{MT}$ are the length and lengthening velocity of the muscular-tendon actuator, respectively. ***L***^*M*^ and ${\dot{L}}^{M}$ are the length and lengthening velocity of the muscle fiber, respectively. ***a*** indicates the muscle activation, ***k***_*p*_ and ***k***_*d*_ are the fb gains, and $L_{0}^{MT}$ and ${\dot{L}}_{0}^{MT}$ are the muscular-tendon actuator length and lengthening velocity for the objective posture, respectively. Each of the symbols listed above represents a 70-dimensional vector.

We developed an NC model (red area in human SPC (Fig 2)) to simulate the biped stance, considering a 120-ms neurological time delay (flesh-colored blocks in Fig 2). A musculoskeletal model was created to approximate the human musculoskeletal system anatomy (green block in Fig 2). The NC model consists of ff and fb controls, and was designed to actuate the muscles in order to hold the musculoskeletal model in an upright standing posture. The ff control output, ***u***_*ff*_, is a set of predetermined constant muscle activations that are used to maintain an objective posture. Details are given in the “Feed-forward control” section. Feedback control is modeled as a PD control incorporating fb information on the muscle length and lengthening velocity.

The large number of variables (53) in the NC model poses a challenge. The variable design process was divided into two stages. The ff control variables were designed in the first stage, after which various ***u***_*ff*_ candidates were collected. Among them, several ***u***_*ff*_ were selected and used to design the fb control variables via an optimization process.

Note that the selected ***u***_*ff*_ must, in conjunction with fb control, simulate muscle activations that reflect physiologically plausible features of the biped stance in order to be considered valid. Therefore, we compared the simulated muscle activations with experimental data to verify which ***u***_*ff*_ could simulate muscle activations that were within the physiologically plausible range.

### Musculoskeletal model

**Fig 3:**
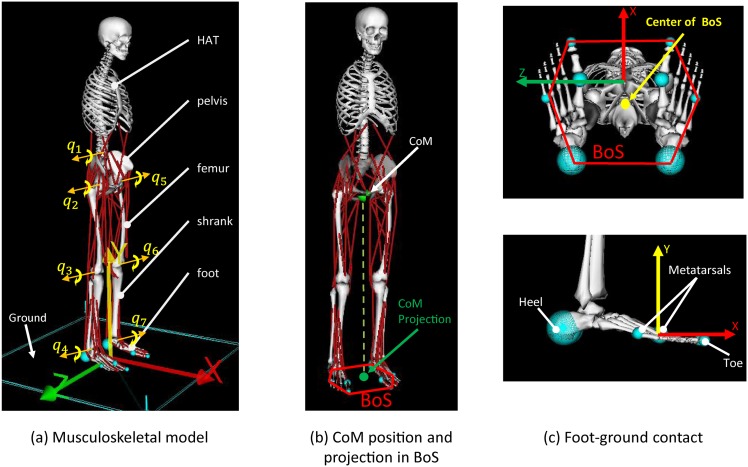
Musculoskeletal model. (a) Musculoskeletal model. HAT: head, arms, and torso. The OpenSim musculoskeletal model consists of seven DoFs and 70 muscular-tendon actuators. *q*_1_–*q*_7_ are the seven joint coordinates, each of which has one DoF for joint flexion and extension. (*q*_1_–*q*_7_: Lumbar, right hip, right knee, right ankle, left hip, left knee, and left ankle flexion-extension, respectively.) (b) CoM position and projection at base of support (BoS). The green-and-black ball is the CoM position, and the green ball is the vertical CoM projection at the BoS. (c) Foot-ground contact. The foot-ground contact is represented by four compliant spheres (heel, toe, and two metatarsals) on each foot, with Coulomb friction.

We created a musculoskeletal model (Fig 3a) in OpenSim 3.3 (SimTK.org) [49], which is an open-source biomechanical simulator. The model is based on the Gait2392 Model [50] and ToyDropLanding Model [51], both of which are provided by the OpenSim 3.3 software, and is designed to simulate the biped stance. The created model is comprised of eight body segments, 70 muscular-tendon actuators, and seven joints, with seven DoFs, as shown in Fig 3a. In this study, we primarily focused on the motion of the lower extremities; therefore, the upper extremities (head, trunk, and arms) are modeled as a single body segment connected to the pelvis by the lumbar joint. The lower extremity section is composed of the femur, shank, and foot, which are connected by the hip, knee, and ankle joints, respectively. As we primarily studied the stance motion in the sagittal plane, all joints are modeled as 1-DoF rotational joints for flexion and extension. The 70 muscular-tendon actuators in the ToyDropLanding Model [51], which are assumed to be important for quiet standing, are employed. These muscles are modeled as 70 Millard 2012 Equilibrium muscular-tendon actuators [52]. Each actuator incorporates a compliant tendon because compliant tendons such as the Achille’s tendon plays an important role in stance postural control [53]. To apply an accurate human musculoskeletal anatomy to the model, the kinematic (e.g., the body segment lengths, joint positions, and muscle attachment points) and dynamic (e.g., the body segment inertial properties, muscle isometric forces, and optimal muscle lengths) information of the Gait2392 Model is used. Note that the Gait2392 Model has rather accurate human anatomy data and has been widely employed in gait analysis [54–57].

The foot-ground contact is modeled as contact between four compliant balls (heel, toe, and two metatarsals in Fig 3c) with a plane (Ground in Fig 3a). The Hunt–Crossley Contact Model [58] defined in the ToyDropLanding Model is employed to calculate the contact force. Specifically, the contact force (ground reaction force) is calculated when the feet begin to penetrate the ground, according to

$$R_{x}=\mu \left(v\right)R_{y},$$ (1)

$$R_{y}=Eh^{\frac{3}{2}}\left(1+\frac{3}{2}b\dot{h}\right),$$ (2)

where *R*_*x*_ and *R*_*y*_ are the frictional force and the vertical ground reaction force, respectively, and *μ*(*v*) is the frictional coefficient function related to the relative velocity *v* between the ground and foot-ground contact point. Note that *μ*(*v*) is defined in terms of *μ*_*s*_, *μ*_*d*_, *μ*_*v*_, and *v*_*t*_, which are the static, dynamic, and viscous friction coefficients and the speed at which the static friction reaches its peak value, respectively. The equation for *μ*(*v*) was defined by Sherman et al. [59]. Further, *E* is the contact stiffness, *h* is the penetration height (the foot soft-tissue deformation displacement), and *b* is the dissipation coefficient. More details on implementing the Hunt–Crossley model in OpenSim are given by Sherman et al. [59].

In this study, *μ*_*s*_, *μ*_*d*_, and *μ*_*v*_ are set to 0.9, 0.9, and 0.6, respectively; *v*_*t*_ is set to 0.1 (m/s), *b* is set to 0.5 (m/s)^−1^, and *E* is set to 10^8^ (Nm^−1.5^). The contact-model variable configuration is determined to avoid unrealistic contact and maintain a reasonable computation speed [59, 60].

### Neurological time delay

In realizing the biped stance, we considered a maximum neurological time delay of 120 ms, which includes a 40-ms feedback delay *τ*_*fb*_, 40-ms transmission delay *τ*_*trans*_, and maximum 40-ms activation dynamics delay *τ*_*act*_. Here, *τ*_*trans*_ is the delay affecting the neural controller’s transmission of the neural controls to the muscle-motion neurons, whereas *τ*_*fb*_ is the delay affecting the sensory receptors’ receipt of the sensory information. Both delays are modeled using a pure time delay of 40 ms, in accordance with Masani et al. [18]. The third delay, *τ*_*act*_, affects the muscle activation dynamics, which is the process through which the muscles generate force following receipt of the control signals. *τ*_*act*_ is 20 or 40 ms, depending on which motion process influences the neurons. The muscle activation dynamics can be divided into activation and deactivation processes, as shown in the breakdown of the activation dynamics delay given in Fig 1. In the activation process, the muscle-motion neurons fire to a higher activation level, as commanded by the neural controller. In contrast, for the deactivation process, the muscle-motion neurons deactivate to a lower activation level. In accordance with Eq (4), *τ*_*act*_ is set to be equivalent to a 20-ms activation time delay *t*_*act*_ during the activation process and a 40-ms deactivation time delay *t*_*dact*_ during the deactivation process. Further, *t*_*act*_ and *t*_*dact*_ are set to 20 and 40 ms, respectively, in accordance with Zajac [61], Winters [62], and Jacobs [63]. The muscle activation dynamics are modeled as a first-order differential equation in OpenSim, with

$$\dot{a_{i}}\left(t\right)=\frac{u_{i}\left(t-\tau_{trans}\right)-a_{i}\left(t\right)}{\delta (a_{i}\left(t\right),u_{i}\left(t-\tau_{trans}\right))},$$ (3)

$$\delta \left(a_{i}\left(t\right),u_{i}\left(t-\tau_{trans}\right)\right)=\left\{\begin{array}{cc} t_{act}\left(0.5+1.5a_{i}\left(t\right)\right), & u_{i}\left(t-\tau_{trans}\right)>a_{i}\left(t\right)\\ t_{dact}/\left(0.5+1.5a_{i}\left(t\right)\right), & u_{i}\left(t-\tau_{trans}\right)\leq a_{i}\left(t\right) \end{array}\right.,$$ (4)

where *u*_*i*_ is the total output from the NC model to the *i*th muscle and *a*_*i*_ represents the muscle activation of the *i*th muscle (*i* = 1, 2, 3, …, 70). *δ* is the delay coefficient.

In this paper, the maximum neurological time delay of 120 ms is referred to as the “120-ms neurological time.”

### Neural controller

Both the ff and fb controls are assumed to be necessary for human SPC. It is widely acknowledged that fb sensory information plays a vital role in postural control [20]; therefore, sensory fb control is indispensable during quiet standing. In addition to fb control, previous physiological studies have indicated the possible existence of ff control [64, 65]. For example, Fitzpatrick et al. [64] suggested that sensory fb control is important, but not sufficient, to stabilize posture in the case of perturbations. Further, Gatev et al. [65] reported that muscle contraction occurs prior to stabilization of the CoM position, implying that ff control may be employed to predict the future CoM position and achieve postural stabilization.

In accordance with the findings of these previous physiological studies [20, 64, 65], our proposed neural controller incorporates ff and fb controls, with outputs labeled ***u***_*ff*_ and ***u***_*fb*_, respectively. Thus, the total output of the NC model, *u*, which is a combination of ***u***_*ff*_ and ***u***_*fb*_, excites the muscle motion neurons to drive the skeletal system, such that

$$u\left(t\right)=u_{ff}+u_{fb}\left(t\right),$$ (5)

where ***u***_*ff*_ and ***u***_*fb*_ are the ff and fb output to 70 muscles. Thus, ***u***, ***u***_*ff*_, and ***u***_*fb*_ are 70-dimensional vectors.

#### Feed-forward control

Unlike the ff control proposed in previous studies [26–30], in this study, ***u***_*ff*_ is the set of activations needed to keep the musculoskeletal model standing in the defined objective posture, i.e., the upright standing posture shown in Fig 3a.

We determine this upright standing posture by tuning *q*_1_, *q*_2_, *q*_4_, *q*_5_, *q*_7_ (Fig 3a) using the Covariance Matrix Adaptation Evolution Strategy (CMA-ES) optimizer available in OpenSim 3.3 (SimTK.org) [49]. Details of the optimizer are described in the “Variable design” section. Note that *q*_3_ and *q*_6_ are fixed to 1°, because we assume that the knees are mildly flexed in the objective posture. The initial solutions for *q*_1_, *q*_2_, *q*_4_, *q*_5_, and *q*_7_ are set to *q*_1_ = −10, *q*_2_ = −5, *q*_4_ = 0, *q*_5_ = −5, and *q*_7_ = 0 for the CMA-ES optimizer. The optimizer generates posture candidates and evaluates their objective function *J*_*pos*_ for each iteration. The optimization terminates when the convergence tolerance reaches the default value defined in the optimizer.

The objective function *J*_*pos*_ that is minimized by the CMA-ES optimizer can be expressed as

$$J_{pos}=w_{static}J_{static}+w_{\Omega }J_{\Omega },$$ (6)

$$J_{static}={\left(CoM_{0,x}-BoS_{x}\right)}^{2}+{\left(CoP_{0,x}-BoS_{x}\right)}^{2},$$ (7)

where *CoM*_0,*x*_ is the vertical projection of the body CoM at the base of support (BoS) (green ball in Fig 3b), *CoP*_0,*x*_ is the center of pressure (CoP) position under each candidate posture. *BoS*_*x*_ is the BoS center (yellow ball in Fig 3c).

*J*_*pos*_ is used to evaluate whether the posture satisfies the static stability conditions. The well-known condition for standing stability in static situations is that the vertical projection of the CoM should be within the BoS [66]. The CoP should also be within the BoS, to ensure that the ground reaction force can be transmitted from the ground to the feet [67]. Hence, both “(*CoM*_0,*x*_ − *BoS*_*x*_)^2^” and “(*CoP*_0,*x*_ − *BoS*_*x*_)^2^” are used to adjust the objective posture, so as to allow both the vertical projection of the CoM and the CoP to be close to the BoS center. This adjustment ensures that the objective posture satisfies the stability condition. Note that the CoP position is not fixed. Rather, the objective-posture CoP position is simply the initial CoP position, which changes with body movement during quiet standing.

There are many posture candidates that satisfy the static condition. To obtain a posture that can be maintained with minimal torque, the following term is also incorporated:

$$J_{\Omega }=\sum_{n=1}^{7}\Omega_{n}^{2},$$ (8)

where Ω_*n*_ is the joint torque necessary for the *n*th joint to maintain this objective posture. We use *w*_*static*_ and *w*_Ω_ to represent the weights of the corresponding terms: *w*_*static*_ is set to 10000, so as to reject any postures that do not satisfy the static stability condition, and *w*_Ω_ is empirically set to 0.1. The specific values of the coordinates for the obtained objective posture are listed in Table 1.

**Table 1:** Coordinate values for objective posture.

Coordinate	Value (deg.)
*q*_1_	12
*q*_2_	6
*q*_3_	1
*q*_4_	-2
*q*_5_	6
*q*_6_	1
*q*_7_	-2

Once the objective posture has been determined, a specific constant muscle activation, *c*_*i*_, which is independent of the fb information, is sent directly to the *i*th muscle. Thus,

$$u_{ff,i}=c_{i},$$ (9)

where *c*_*i*_ is the *i*th element of ***u***_*ff*_, and ensures that the *i*th muscle maintains balance in the objective posture (*i* = 1, 2, 3, …, 70).

The ***u***_*ff*_ components compensate for gravity and increase the joint stiffness resulting from muscle contraction. Note that human joint stiffness contributes to balance during SPC. In this paper, two sources of joint stiffness are considered: the passive mechanical source (tendons), which is modeled as a spring-like tendon component in a muscular-tendon actuator [52], and the active source, called active stiffness, resulting from ***u***_*ff*_ . ***u***_*ff*_ is similar, but not identical, to muscle co-contraction, which stimulates the activation of antagonist muscles around a joint so as to fixate that joint. Muscle co-contraction increases the joint stiffness only. The net torque generated by muscle co-contraction is zero; therefore, it does not contribute to gravitational compensation. However, the net torque generated via ***u***_*ff*_ compensates for the gravitational torque.

In a previous muscle co-contraction study [68], the active stiffness level was typically quantified based on the muscle activation level. Similarly, in this paper, the square norm of ***u***_*ff*_, ||***u***_*ff*_|| ($\left|\right|u_{ff}\left|\right|=c_{1}^{2}+c_{2}^{2}+c_{3}^{2}+...+c_{i}^{2}$), is used to quantify the active stiffness level.

#### Feedback control

Sensory information is critical for realizing the biped stance. Human SPC is realized via an fb mechanism that actuates muscles to generate appropriate corrective torques, so as to counter the destabilizing torque due to gravity [8]. The corrective torques are generated through the integration of multisensory inputs, including the visual, vestibular, and proprioceptive somatosensory inputs.

Proprioceptive somatosensory input is assumed to make the largest contribution to the SPC during quiet standing. Winter et al. reported that vestibular input may not make a significant contribution to the control of the upright stance [3], and Sousa et al. argued that, as in normal conditions, proprioceptive information has more relevance than other sensory sources. Hence, we employed the proprioceptive sensory input as fb information only, despite the existence of multisensory inputs [69].

The fb control is approximated by a PD controller (Eq (10)). This controller functions based on proprioceptive sensory information concerning the muscular-tendon length and lengthening velocity. Thus,

$$u_{fb,i}\left(t\right)=k_{p,i}\left(\frac{{L_{i}}^{MT}\left(t-\tau_{fb}\right)-L_{0,i}^{MT}}{L_{0,i}^{MT}}\right)+k_{d,i}\left(\frac{{{\dot{L}}_{i}}^{MT}\left(t-\tau_{fb}\right)-{\dot{L}}_{0,i}^{MT}}{{V_{i}}^{max}}\right),$$ (10)

where ${V_{i}}^{max}$ is the maximum muscle lengthening velocity, ${L_{i}}^{MT}\left(t-\tau_{fb}\right)$, and ${\dot{L_{i}}}^{MT}\left(t-\tau_{fb}\right)$ are the delayed muscular-tendon length and lengthening velocity, respectively, $L_{0,i}^{MT}$ is the muscular-tendon length reference, which is the length for the objective posture, and $L_{0,i}^{MT}$ is the lengthening velocity reference. Further, *k*_*p*,*i*_ and *k*_*d*,*i*_ are the PD gains of the *i*th muscle (*i* = 1, 2, 3, …, 70). ${\dot{L}}_{0,i}^{MT}$ is set to 0 *m*/*s* in order to approach a stable stance. Note that this controller is different from stretch reflex control which adopts muscle fiber length as feedback information. Instead, muscular-tendon length, sum of muscle length and tendon length, is adopted as feedback information. The muscle fiber length moves in the opposite direction of human body. For example, when musculoskeletal model moves forward, soleus muscle fiber length contract to generate the bias of the tendon and torque to move the musculoskeletal model backward. This paradoxical muscle movement characteristics coincides with that reported in previous study [53].

#### Variables in neural controller

In total, the neural controller has 210 variables, including a 70-dimensional ***u***_*ff*_ (70 constant *c* values in Eq (9)) and 140 PD gains (70 proportional *k*_*p*_ and 70 derivative *k*_*d*_ gains, Eq (10)). In this paper, we mainly focus on the anterior-posterior body movement in the sagittal plane during quiet standing (joint displacements are symmetrical: *q*_2_ = *q*_5_, *q*_3_ = *q*_6_, and *q*_4_ = *q*_7_). Further, we assume that muscles can be grouped based on their function on the joints. Hence, the following assumption-based simplifications are employed:

1. The control laws for the ff and fb control on the muscles are taken to be symmetrical (e.g., the left and right soleus have the same *c* and the same *k*_*p*_ and *k*_*d*_);
2. Muscles located around the same joint and having similar functions on the joint are assumed to have the same PD gains (e.g., the pectineus and psoas are taken to have the same PD gains, because they are both positioned around the hip and have hip flexion functionality).

Specifically, we divide the 70 muscular-tendon actuators into nine groups in accordance with the muscle functions and attachment point positions, obtaining the lumbar extensor, lumbar flexor, hip extensor, hip flexor, knee extensor, knee flexor, ankle extensor, ankle flexor, and biarticular muscle groups. The biarticular muscle group is introduced because the biarticular muscles generate torque on two joints and may have different effects on those joints. The *k*_*p*_ and *k*_*d*_ of each muscle are not unique, and members of the same muscle group have the same values for these fb gains. Thus, the gain of each muscle is dependent on its muscle group. For example, the pectineus and psoas are assigned to the hip extensor group, and both have fb gains of [*k*_*p*_*l*_*ex*_, *k*_*d*_*l*_*ex*_]. The following assignments are made:

$$\left[k_{p,i},k_{d,i}\right]=\left\{\begin{array}{cc} {}[k_{p\_l\_ex},k_{d\_l\_ex}], & \;\text{group}\;=\;\text{lumbar}\;\text{extensor},\\ {}[k_{p\_h\_ex},k_{d\_h\_ex}], & \;\text{group}\;=\;\text{hip}\;\text{extensor},\\ {}[k_{p\_k\_ex},k_{d\_k\_ex}], & \;\text{group}\;=\;\text{knee}\;\text{extensor},\\ {}[k_{p\_a\_ex},k_{d\_a\_ex}], & \;\text{group}\;=\;\text{ankle}\;\text{extensor},\\ {}[k_{p\_l\_fl},k_{d\_l\_fl}], & \;\text{group}\;=\;\text{lumbar}\;\text{flexor},\\ {}[k_{p\_h\_fl},k_{d\_h\_fl}], & \;\text{group}\;=\;\text{hip}\;\text{flexor},\\ {}[k_{p\_k\_fl},k_{d\_k\_fl}], & \;\text{group}\;=\;\text{knee}\;\text{flexor},\\ {}[k_{p\_a\_fl},k_{d\_a\_fl}], & \;\text{group}\;=\;\text{hip}\;\text{flexor},\\ {}[k_{p\_bi},k_{d\_bi}], & \;\text{group}\;=\;\text{biarticular}\;\text{muscle}, \end{array}\right.$$ (11)

where “group” indicates the muscle group.

The muscle gains are grouped for muscles in the same group, and each member of a given group has the same PD gains but different *c*. Note that the ***u***_*ff*_ inputs are not grouped, because ***u***_*ff*_ is added to the muscles directly without any fb information. It is difficult to balance the net torque generated by the extensors and flexors, as each muscle has unique muscle properties. Muscle elongation can actually differ among muscles of the same group because of their different moment arm. We assume that moment arm is approximately constant during quiet standing. In general, the muscle fiber length and muscle moment arm are positively correlated [70]. Hence, in Eq (10), we use $L_{0,i}^{MT}$ to normalize muscular-tendon length feedback information to reduce the effect of moment arm. We also normalize the velocity information by $V_{i}^{M}T$ to obtain dimensionless derivative gains. This normalization allows muscles in the same group to be controlled by the same gains. As a result of the normalization, [*k*_*p*,*i*_, *k*_*d*,*i*_] is dimensionless.

Thus, the number of essential variables for our proposed neural controller design has been reduced from 210 to 53, including a 35-dimensional ***u***_*ff*_ and 18 PD gains (as listed in Eq (11)).

### Variable design

**Fig 4:**
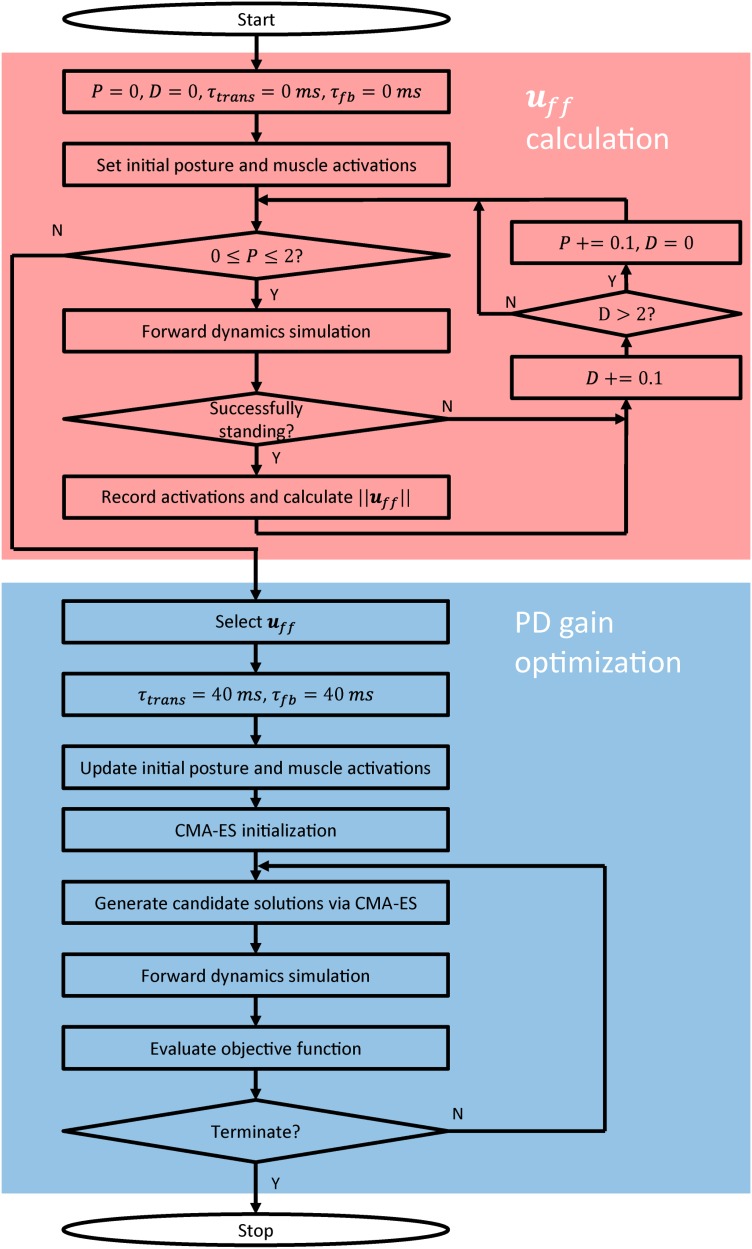
Variable design algorithm. The red and blue areas indicate the ***u***_*ff*_ calculation algorithm and the PD gain optimization algorithm, respectively.

As explained above, 53 variables are required to model the controller. This is very challenging as regards determining a suitable solution for many variables to keep a musculoskeletal model standing under the influence of the 120-ms neurological time delay. As ***u***_*ff*_ is constant and independent of the neurological time delay, the various ***u***_*ff*_ candidates (the 35 constant *c* values in Eq 9) are first determined (Fig 4, red area); *τ*_*trans*_ and *τ*_*fb*_ are both 0 ms for these variables. Details of the calculation are given in the “***u***_*ff*_ calculation” section. Among the various calculated ***u***_*ff*_ candidates, several are selected based on the value of ||***u***_*ff*_||. Subsequently, for each selected ***u***_*ff*_, the PD gains are optimized (Fig 4, blue area) so as to maintain the standing posture under the influence of the 120-ms neurological time delay.

#### *u*_*ff*_ calculation

This section describes the calculation of the many 35-dimensional ***u***_*ff*_ candidates (the *c* values in Eq (9)) needed to hold the musculoskeletal model in an objective standing posture (Table 1).

To calculate ***u***_*ff*_, which is independent of the neurological time delay, the muscle activations during a stable biped stance in the objective posture must be obtained, with *τ*_*trans*_ and *τ*_*fb*_ both set to 0 ms. The obtained muscle activations define ***u***_*ff*_, and can be used as the ***u***_*ff*_ input. A PD controller receives the same feedback information but different gain types in Eqs (10) and (11) is used to generate a biped stance in the objective standing posture. The desired muscle activations are then obtained by calculating the integrated muscle activations over the stable stance period (Eq (15)). These are used as the ***u***_*ff*_ input.

Note that the PD controller above is only used to calculate ***u***_*ff*_, and is not related to the PD controller that copes with feed-forward control to compensate for the 120-ms neurological time delay used in the following “PD gain optimization” section.

To obtain physiologically plausible ***u***_*ff*_, the gains of the flexors and biarticular muscles are scaled with respect to the extensors. We allow all of the flexors and extensors to have identical gains, and scale the gains of the flexor and biarticular muscles by factors of 0.5 and 0.2, respectively, with respect to those of the extensors. The scaling of PD gains is based on the physiological knowledge that the extensors are the dominant mechanism during quiet standing, whereas the biarticular muscles contribute little to this posture [71]. Therefore, we assume that the extensors have larger gains than the flexors and biarticular muscles, and that the biarticular muscles have the lowest gains. Thus, the gains in Eq (11) can be described by *P* and *D* as follows:

$$k_{p\_l\_ex}=k_{p\_h\_ex}=k_{p\_k\_ex}=k_{d\_a\_ex}=P,k_{p\_l\_ex}=k_{d\_h\_ex}=k_{d\_k\_ex}=k_{d\_a\_ex}=D,$$ (12)

$$k_{p\_l\_fl}=k_{p\_h\_fl}=k_{p\_k\_fl}=k_{d\_a\_fl}=0.5P,k_{p\_l\_fl}=k_{d\_h\_fl}=k_{d\_k\_fl}=k_{d\_a\_fl}=0.5D,$$ (13)

$$k_{p\_bi}=0.2P,k_{d\_bi}=0.2D,$$ (14)

where *P* and *D* are the values of the extensor proportional gain and derivative gain, respectively. Note that the scaling of gains is only conducted for the ***u***_*ff*_ calculation, and *P* and *D* are the only variables in the ***u***_*ff*_ calculation.

To calculate various ***u***_*ff*_ candidates, as shown in Fig 4 (red area), we conduct an extensive search for the *P* and *D* values needed to maintain a standing posture for 60 s, when *τ*_*trans*_ and *τ*_*fb*_ are both 0 ms, using a forward dynamics simulation. Note that *τ*_*act*_ is not neglected, because the muscle dynamics must be incorporated to obtain physiologically plausible muscle functionality. The simulation end time was set to 60 s because this is assumed to be sufficiently long to allow the musculoskeletal model to achieve a stable stance state. The initial posture was set to the same as the objective posture (Table 1), and the initial muscle activations *a*_*i*_(*t*)|_*t* = 0_ were set to zero.

We search for *P* and *D* in the range 0.0–2.0 at increments of 0.1. The muscle activations are only recorded if the musculoskeletal model is capable of standing (i.e., if the CoM was higher than 0.4 m). The integration of the muscle activations when the musculoskeletal model achieves a stable standing posture is adopted as ***u***_*ff*_. In other words, the *c*_*i*_ in Eq (9) are calculated according to

$$u_{ff,i}=c_{i}=\frac{\int_{t_{1}}^{t_{2}}a_{i}\left(t\right)dt}{t_{2}-t_{1}},$$ (15)

where *t*_1_–*t*_2_ is the period for which the musculoskeletal model maintains a stable posture (*t*_1_ = 3 s and *t*_2_ = 5 s).

||***u***_*ff*_|| is then computed to quantify the active stiffness level.

#### PD gain optimization

Among the various ***u***_*ff*_ candidates obtained as described above, we select several based on ||***u***_*ff*_||. For each selected ***u***_*ff*_, the PD gain variables are designed based on an optimization procedure (Fig 4, blue area) to cope with the selected ***u***_*ff*_ and compensate for the 120-ms neurological time delay. CMA-ES is employed to optimize the 18 PD gain variables that act to hold the musculoskeletal model in a standing posture for 60 s, using a forward dynamics simulation that incorporates the selected ***u***_*ff*_ along with the 40-ms *τ*_*trans*_ and *τ*_*fb*_. The initial posture is updated to the stable posture that can be maintained with the selected ***u***_*ff*_, and the initial *a* values are updated in accordance with ***u***_*ff*_ (*a*_*i*_(*t*)|_*t* = 0_ = *u*_*ff*,*i*_). CMA-ES is an evolution algorithm for solving nonlinear black-box optimization problems [72, 73], and has been successfully app lied by Dorn et al. [60] to optimize a complicated controller for gait generation. This algorithm does not calculate the gradient of the objective function but, rather, estimates the covariance matrix. The variables are the population size *λ*, initial standard deviation *σ*, and the initial solution and termination criteria. In this study, the optimizer was initialized by setting *λ* = 20 and *σ* = 0.005 for fast convergence. The initial solution was generated from a seed. In addition to the default termination criteria, a maximum iteration number of 750 was defined. The simulation was conducted so as to evaluate 20 candidate solutions generated by the CMA-ES in parallel in each iteration. The number of child threads created for parallel execution is equivalent to the number of computer cores.

CMA-ES is used to minimize the objective function *J*, where

$$J=w_{fail}J_{fail}+w_{stability}J_{stability},$$ (16)

$$J_{fail}=\frac{T_{simu}-T_{fail}}{T_{fail}},$$ (17)

with *T*_*simu*_ = 5 s being the simulation end time and *T*_*fail*_ being the time at which the musculoskeletal model begins to collapse or the heel or toe leave the ground. This event is monitored by an event trigger that terminates the simulation when the height of the CoM is less than 0.4 m, or when the heel or toe contact force is 0 *N*. *T*_*fail*_ is the time at which this event occurred. Note that, if the failure event did not occur until *T*_*simu*_, then *T*_*fail*_ = *T*_*simu*_ and *J*_*fail*_ = 0. The event trigger decreases the computation time of the optimization during the early iterations. *J*_*stability*_ is used to evaluate the stability of the biped stance and the deviation of the posture from the objective posture. This term is expressed as

$$J_{stability}=\sum_{n=1}^{7}\int_{0}^{T_{simu}}\left|q_{n}\left(t\right)-q_{n}\left(0\right)\right|dt,$$ (18)

where *q*_*n*_(*t*) is the *n*th coordinate value (angle displacement of one joint DoF) and *q*_*n*_(0) is the *n*th initial coordinate value.

The failure weight, *w*_*fail*_, was set to 500 000 in order to reject any solutions in which the musculoskeletal model failed to stand. The stable weight, *w*_*stability*_, was set to 50 so as to rapidly discover solutions that hold the musculoskeletal model in a stable standing position and in a posture close to the objective. The values of 500 000 and 50 were determined based on a study reported by Dorn et al. [60]. In addition, if $J_{fail}^{2}$ or $J_{stability}^{2}$ is smaller than a minimum value (1.0*e*^−6^), they will be set to 0.

### Evaluation of simulated results

To evaluate our simulated results, we compared them with experimental data, including the CoM anterior-posterior (AP) displacement range, CoP AP displacement range, joint correlations and, the muscle activation range for a human biped stance [71, 74].

To investigate which selected ***u***_*ff*_ could function with the fb control to generate physiologically plausible muscle activations, we compared the simulated muscle activations against the experimental muscle activation range [71] to determine whether the simulated activations were in the range of the experimental data. Further, the deviation of the simulated muscle activations from the range data was calculated. Experimental muscle activation range and mode value data were reported by Panzer et al. [71], who studied 24 normal subjects, including 12 young subjects (age: 21–57 years; mean age: 38.4 years) and 12 elderly subjects (age: 63–77 years; mean age: 68.1 years). In that study, the subjects stood in an erect posture on a platform with a fre ely chosen foot position. Electromyographic (EMG) data normalized by the maximal voluntary contraction (MVC) of eight muscles were collected.

Further, to investigate the physiologically plausibility of postural sway patterns, we evaluated the following two aspects: 1. CoM AP and CoP displacement trajectory, and 2. multi-joint coordination. We plotted the CoM AP and CoP displacement trajectory. The CoM and CoP AP range are used as indicators to evaluate the physiological plausibility. The simulated CoM and CoP AP displacement range were compared against experimental data reported by Warnica et al. [74], who studied 16 young adults (whose age, height, and body mass (mean (SD)) were 22.6(1.4) years, 173.3(11.1) cm, and 70.7(12.9) kg, respectively). In that study, CoM and CoP displacement data for each adult were measured during quiet standing, and then the CoM and CoP AP displacement range were calculated and analyzed. We calculated the hip-ankle, hip-knee, and knee-ankle angle correlation coefficient, and compared against experimental data [43] to evaluate the multiple-joint coordination.

In addition, to evaluate whether the generated biped stance motion was stable, the CoM AP displacement versus CoM AP velocity was plotted.

## Results

### Variable design

We conducted an extensive search for ***u***_*ff*_ candidates, and obtained a total of 402 ***u***_*ff*_ that successfully held the musculoskeletal model in a standing posture. Among them, we selected nine ***u***_*ff*_ based on ||***u***_*ff*_|| (||***u***_*ff*_|| = 0.06, 0.89, 2.07, 3.44, 4.00, 5.04, 6.00, 7.00, and 8.02), and conducted further PD gain optimization with a neurological time delay of 120 ms. The PD gains were optimized for each ***u***_*ff*_ using the CMA-ES algorithm. As a result (Table 2), the gains for all ***u***_*ff*_ (except ||***u***_*ff*_|| = 0.06) were found to successfully maintain the musculoskeletal model in a standing posture (S1 Video).

**Table 2:** Variable design results.

||*u*_*ff*_||	*k*_*p*_*l*_*ex*_	*k*_*d*_*l*_*ex*_	*k*_*p*_*h*_*ex*_	*k*_*d*_*h*_*ex*_	*k*_*p*_*a*_*ex*_	*k*_*d*_*a*_*ex*_	*k*_*p*_*k*_*ex*_	*k*_*d*_*k*_*ex*_	*k*_*p*_*l*_*fl*_	*k*_*d*_*l*_*fl*_	*k*_*p*_*h*_*fl*_	*k*_*d*_*h*_*fl*_	*k*_*p*_*a*_*fl*_	*k*_*d*_*a*_*fl*_	*k*_*p*_*k*_*fl*_	*k*_*d*_*k*_*fl*_	*k*_*p*_*bi*_	*k*_*d*_*bi*_
0.89	0.055	0.058	0.040	0.025	0.136	0.083	0.053	0.089	0.094	0.002	0.029	0.002	0.102	0.120	0.143	0.009	0.117	0.007
2.07	0.050	0.077	0.050	0.003	0.289	0.056	0.046	0.246	0.020	0.007	0.131	0.001	0.057	0.112	0.108	0.027	0.149	0.023
3.44	0.098	0.077	0.068	0.002	0.198	0.126	0.024	0.157	0.111	0.001	0.092	0.009	0.038	0.023	0.067	0.105	0.223	0.037
4.00	0.113	0.048	0.066	0.017	0.092	0.177	0.080	0.138	0.128	0.012	0.014	0.054	0.127	0.013	0.525	0.001	0.268	0.006
5.04	0.118	0.035	0.097	0.018	0.100	0.057	0.163	0.087	0.173	0.039	0.002	0.004	0.097	0.061	0.089	0.040	0.254	0.019
6.00	0.011	0.179	0.096	0.001	0.239	0.037	0.210	0.066	0.108	0.071	0.007	0.002	0.267	0.058	0.073	0.105	0.059	0.001
7.00	0.283	0.108	0.023	0.008	0.166	0.062	0.151	0.027	0.215	0.030	0.026	0.006	0.367	0.149	0.297	0.023	0.106	0.010
8.02	0.350	0.112	0.014	0.025	0.199	0.008	0.133	0.021	0.257	0.021	0.027	0.003	0.503	0.144	0.154	0.145	0.133	0.008

### Evaluation of simulated results

**Fig 5:**
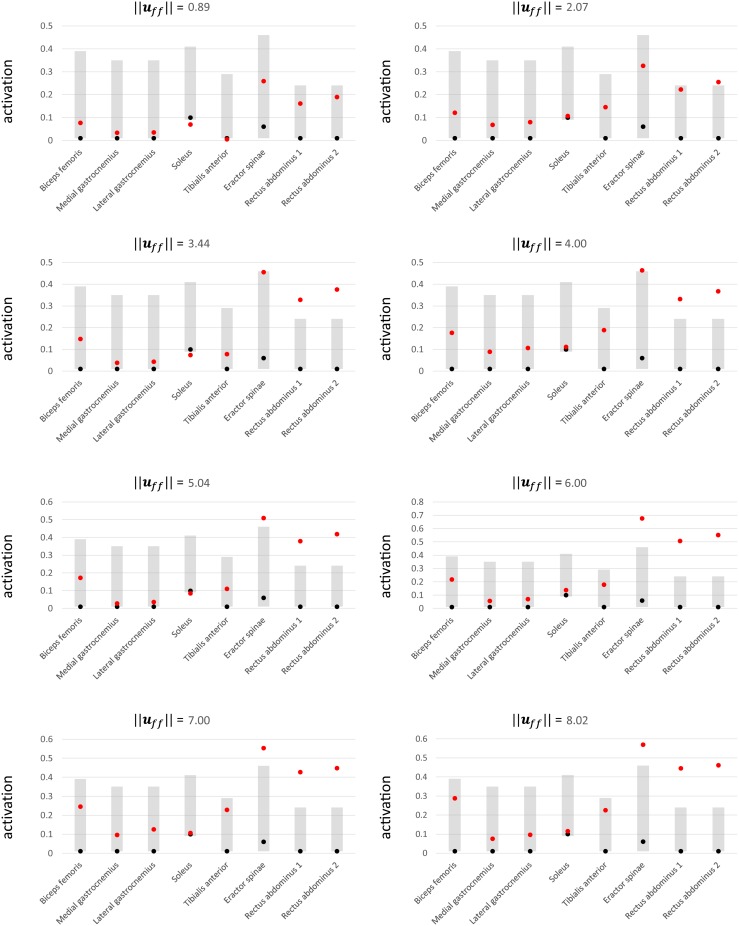
Simulated muscle activations. The red dots are simulated activations, the black dots are mode values, and the gray shaded boxes indicate the experimental activation data ranges. The experimental mode values and activation data ranges were reported in [71].

To investigate which ***u***_*ff*_ could function with the PD control to generate physiologically plausible muscle activations, we compared the simulated muscle activations (red dots in Fig 5) against the experimental muscle activation range (gray shaded boxes in Fig 5). The deviations of the simulated activations from the range data are listed in Table 3. The muscle activations when ||***u***_*ff*_|| = 2.07 were generally within the range of the experimental data. Only the result for rectus abdominus 2 was slightly higher (0.025) than the higher limit of the experimental data range. As for the other ||***u***_*ff*_||, two muscles fell outside the experimental range for ||***u***_*ff*_|| = 0.89, three muscles fell outside the experimental range for ||***u***_*ff*_|| = 4.00, 6.00, 7.00, and 8.02, and four muscles fell outside the experimental range for ||***u***_*ff*_|| = 3.44, and 5.04.

**Table 3:** Deviations from value ranges.

		||*u*_*ff*_||							
		0.89	2.07	3.44	4.00	5.04	6.00	7.00	8.02
**Muscle**
	**Biceps femoris**	0.000	0.000	0.000	0.000	0.000	0.000	0.000	0.000
	**Medial gastrocnemius**	0.000	0.000	0.000	0.000	0.000	0.000	0.000	0.000
	**Lateral gastrocnemius**	0.000	0.000	0.000	0.000	0.000	0.000	0.000	0.000
	**Soleus**	-0.020	0.000	-0.016	0.000	-0.005	0.000	0.000	0.000
	**Tibialis anterior**	-0.005	0.000	0.000	0.000	0.000	0.000	0.000	0.000
	**Eractor spinae**	0.000	0.000	0.005	0.014	0.060	0.228	0.103	0.120
	**Rectus abdominus 1**	0.000	0.000	0.099	0.101	0.149	0.278	0.196	0.216
	**Rectus abdominus 2**	0.000	0.025	0.145	0.137	0.189	0.322	0.217	0.232
**Number of muscles falling outside of range**		2	1	4	3	4	3	3	3

The “0.000” values indicate that the simulated activation is within the range of the corresponding experimental data value. Negative and positive values indicate the deviation of the simulated activation from the lower and upper limits of the range, respectively.

**Fig 6:**
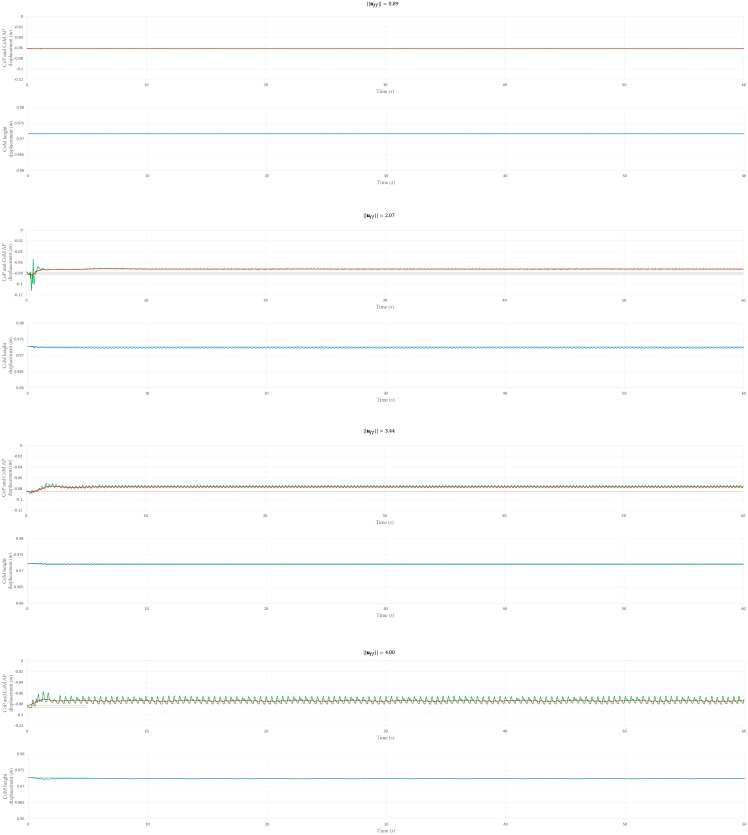
CoP AP, CoM AP, and CoM height displacement for ||*u*_*ff*_|| = 0.89, 2.07, 3.44, and 4.00. The green, red, and blue solid lines are the CoP AP, CoM AP, and CoM height displacements, respectively. The green, red, and blue dotted lines are the CoP position, CoM horizontal position, and CoM height position values for the objective posture, respectively. The positive direction of the “CoP and CoM AP displacement (m)” axis represents the anterior direction.

**Fig 7:**
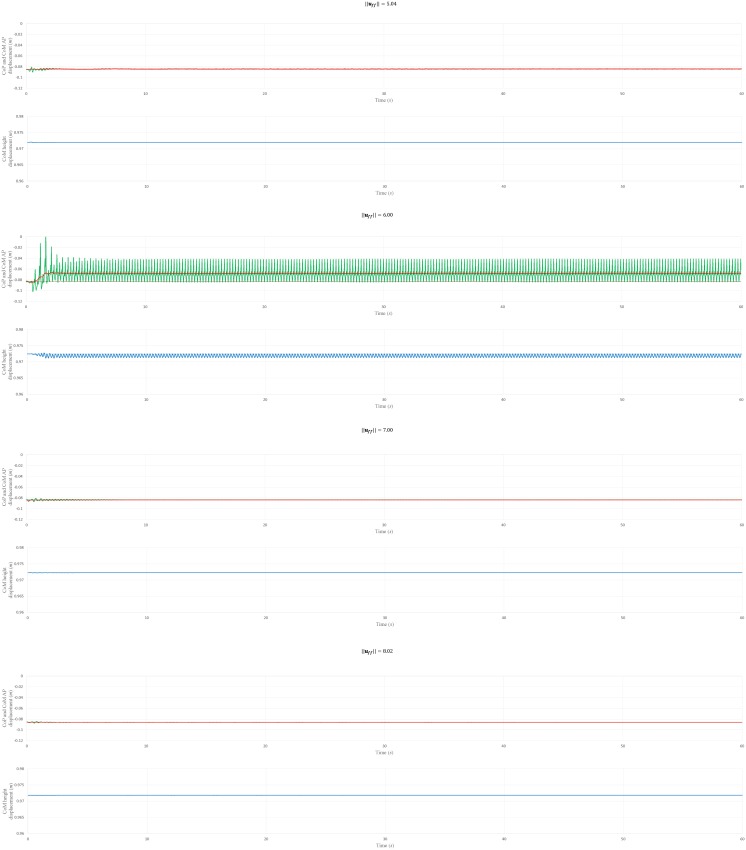
CoP AP, CoM AP, and CoM height displacement for ||*u*_*ff*_|| = 5.04, 6.00, 7.00, and 8.02. The green, red, and blue solid lines are the CoP AP, CoM AP, and CoM height displacements, respectively. The green, red, and blue dotted lines are the CoP position, CoM horizontal position, and CoM height position value for the objective posture, respectively. The positive direction of the “CoP and CoM AP displacement (m)” axis represents the anterior direction.

To investigate the physiological plausibility of postural sway patterns during a 60-s simulation, the CoM AP, CoP AP, and CoM height displacements were plotted, as shown in Figs 6 and 7. The CoM oscillates around a stable state value, and the CoP oscillates around the CoM trajectory. From the CoM height displacement, it was determined that all the selected ***u***_*ff*_ allowed the musculoskeletal model to maintain a standing position via a periodical height sway. In addition, the CoM AP and CoP AP displacement ranges (maximum displacement minus minimum displacement over 30–60 s, which is the period in which the musculoskeletal model achieved a stable-stance state) were obtained; see Table 4. We compared the ranges with those reported by Warnica et al. [74], who reported the mean±SD of CoP AP and CoM AP to be 20.48±6.97 mm and 17.36±5 mm, respectively. We confirmed that the simulated CoM AP had a smaller range for all ||***u***_*ff*_||, whereas the CoP AP range for all ||***u***_*ff*_|| except ||***u***_*ff*_|| = 6.00 had a smaller range than those of a human.

**Table 4:** CoP and CoM AP displacement ranges.

||*u*_*ff*_||	CoP AP range (10^−3^ *m*)	CoM AP range (10^−3^ *m*)
0.89	0.18	0.01
2.07	1.60	0.26
3.44	4.51	0.25
4.00	15.55	2.40
5.04	0.55	0.18
6.00	43.33	1.30
7.00	0.06	0.04
8.02	0.22	0.11

The CoP and CoM displacement ranges are the differences between the maximum and minimum displacements over the period 30–60 s, which corresponds to the period in which the musculoskeletal model achieved a stable stance.

Note that the CoM AP, CoP AP, and CoM height values for the objective posture (the dotted line in Fig 6) differed for each ||***u***_*ff*_||. This is because the objective posture was updated after each ***u***_*ff*_ calculation. That is, the same objective posture was employed in each ***u***_*ff*_ calculation; however, the final stable posture (which was used as the objective posture during the PD gain optimization) was different, because of the muscle force-generation capability. The muscles worked to realize the objective posture for the musculoskeletal model; however, the maximum isometric force rendered the generation of sufficient force difficult and, as a result, the muscles could only maintain a reasonably similar posture to the objective.

In addition, joint correlation coefficients were calculated as shown in Table 5. when ||***u***_*ff*_|| < 7.00, hip-ankle angle are negatively correlated, whereas the knee-ankle angle are positively correlated. This result fits the experimental data that hip and ankle angle are negatively correlated (-0.91±0.054); Both in-phase (0.88±0.000) and anti-phase (-0.87±0.054) of correlations between ankle and knee were observed [43]. However, hip-knee correlation are anti-phase when ||***u***_*ff*_|| = 0.89, 3.44, and 6.00, which differs from the experimental data that hip and knee exhibit a positive correlation (0.89±0.053) [43]. When ||***u***_*ff*_|| = 7.00 and 8.02, the hip-ankle angle has positive correlation, whereas both the hip-knee and knee-ankle angle are negatively correlated. In this case, musculoskeletal model has high overall joint stiffness and sways like an inverted pendulum. The rotations of the hip, knee and ankle all contribute to move CoM in the same direction, i.e., the hip and ankle flex, and the knee extend to move CoM backward.

**Table 5:** Joint angular correlations.

||*u*_*ff*_||	hip-knee	hip-ankle	knee-ankle
0.89	-0.452	-0.913	0.768
2.07	0.363	-0.579	0.373
3.44	-0.458	-0.902	0.767
4.00	0.617	-0.461	0.061
5.04	0.125	-0.504	0.328
6.00	-0.927	-0.876	0.937
7.00	-0.988	0.992	-0.985
8.02	-0.648	0.609	-0.736

**Fig 8:**
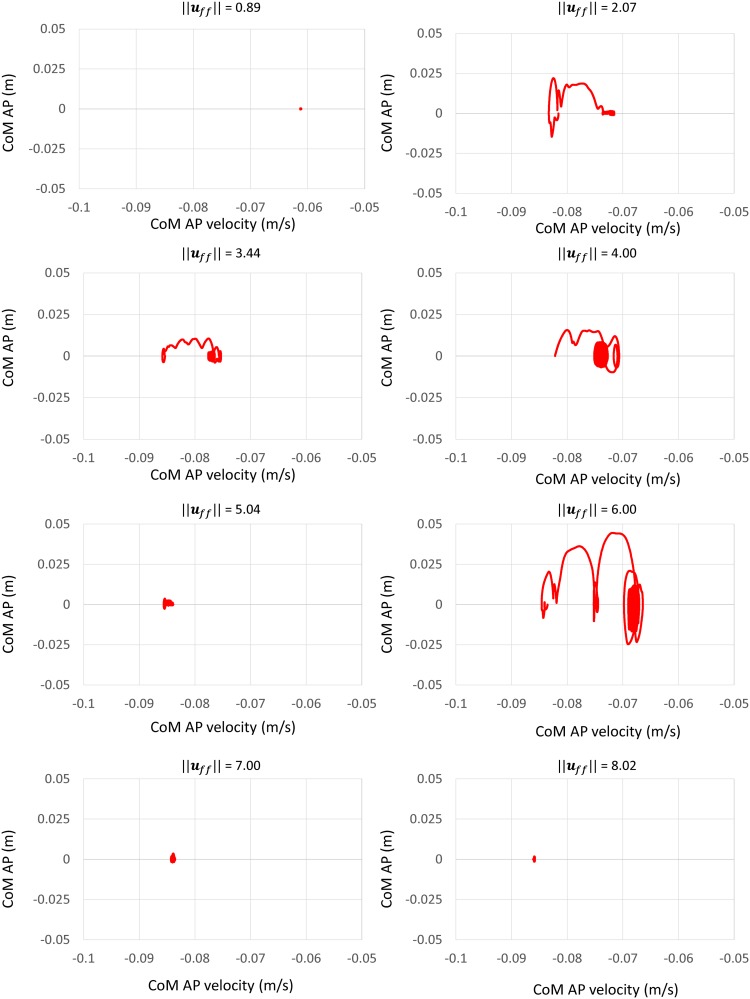
CoM AP displacement vs. CoM AP velocity.

To evaluate whether the generated biped stance motion was stable, CoM AP displacement versus CoM AP velocity was plotted, as shown in Fig 8. As shown in the figure, the attractor of each ||***u***_*ff*_|| is a limit cycle, which indicates that all of the ||***u***_*ff*_|| capable of keeping the musculoskeletal model standing stably. The CoM oscillation may result from the non-linear dynamics of the system (e.g. muscular-tendon dynamics).

## Discussion

Our first goal was to develop an NC model capable of compensating for a 120-ms neurological time delay, so as to allow a musculoskeletal model to stand. The postural control model proposed in this study is the first to have been reported to successfully maintain a musculoskeletal model in a standing posture for the case of a physiologically plausible human anatomy (multiple joints, 70 muscles, and human-like skeletal inertial and muscle dynamics properties) and a 120-ms neurological time delay. In previous studies, researchers have generally employed an inverted pendulum model to investigate the delay compensation mechanism; however, such a model cannot be used to investigate the muscle activation contributions. Hence, we replaced this simplified human model with a human-like musculoskeletal model. Our simulation results indicate that the ***u***_*ff*_, in conjunction with the fb control, can compensate for the neurological time delay. In this study, the fb control employed proprioceptive sensory information only, because this was assumed to have more relevance than other sensory data such as visual and vestibular inputs during quiet standing [69]. However, Peterka has reported that multisensory inputs are important for humans to perform a task, and the contributions of vestibular sensory inputs increase with an increase in the level of external disturbance [7]. The present simulation results indicate that, for an unperturbed stance, a certain ||***u***_*ff*_|| functioning with proprioceptive sensory feedback can compensate for the loss of visual and vestibular sensory input. For the case of a perturbed stance, however, we believe that other sensory input should be incorporated in order to counter the external disturbance. Further, an estimation and anticipation mechanism [20, 30], which would estimate the disturbance acting on the human body and “the internal model” (usually the human body orientation and posture), may also be necessary to counter such a disturbance.

Our second goal was to develop an NC model capable of generating a human-like stance and exhibiting the salient features of the human biped stance. One feature reflected by our NC model is physiologically plausible simulated muscle activations. In particular, the muscle activations for ||***u***_*ff*_|| = 2.07 are considered to be physiologically plausible activations for the human biped stance. That is, the majority of the muscle activations for ||***u***_*ff*_|| = 2.07 were within the physiologically plausible range, based on data obtained via an experimental study, although the activation result for rectus abdominus 2 was slightly higher than the experimental data range. Note that rectus abdominus 2 has higher activations because the functions of all the muscles surrounding the lumbar that contribute to lumbar flexion are condensed into only two lumbar flexors (rectus abdominus 1 and rectus abdominus 2) in the musculoskeletal model. Hence, rectus abdominus 1 and rectus abdo minus 2 require slightly elevated activations to generate the forces that are actually produced by all of the lumbar flexors. The second feature reflected by the NC model is that humans employ a strategy involving a low muscle active stiffness during quiet standing, so as to achieve low energy consumption. The present simulation results indicate that human beings may be capable of standing using various active stiffness levels. ||***u***_*ff*_|| = 2.07 is a physiologically plausible active stiffness level, and is low compared with the highest level (||***u***_*ff*_|| = 8.02) among the selected ***u***_*ff*_. Therefore, for a normal person standing with a relaxed posture, a low active stiffness level may be sufficient to compensate for the neurological time delay and maintain a standing posture. This coincides with the well-known physiological result that humans select a low active stiffness level during quiet standing to reduce energy consumption.

The two features discussed above coincide with current physiological knowledge on the human biped stance; therefore, our proposed NC model and variable design framework successfully generated a physiologically plausible human-like biped stance and may be used in various potential applications, such as to assist with device development and design robotic control systems.

Furthermore, the NC model can also partly represent the characteristics of sway patterns. The postural sway patterns observed when musculoskeletal model stands under physiologically plausible muscle activations (||***u***_*ff*_|| = 2.07) partly resemble the features of that of human beings. The hip and ankle angle are negatively correlated; Both hip-knee and knee-ankle angle have a positive correlation (Table 5). This result coincides with the correlation coefficient obtained from experimental data [43]. However, the simulated CoM AP and CoP AP ranges are smaller than those indicated by the experimental data. This difference is likely to be because the sensory noise level, which is assumed to be one of the variants accounting for the postural sway [75], is not incorporated in the fb control. The sensory noise may induce larger CoM AP sway range, as well as higher anti-phase coupling of the hip and the ankle to maintain the balance. In addition, the neglect of factors such as age and heart rate may have caused the smaller postural sway. However, it would be difficult to develop an NC model that incorporates all the factors that influence postural sway. In this study, we primarily focused on whether or not the employed NC model could generate muscle activations for a stable biped stance. The NC model utilized in this study provides a foundation for the development of a more complex NC model, which could reflect more physiologically plausible features such as postural sway.

## Limitations and Future Work

One limitation of our study is that some rational simplifications were made to the PD controller. More gains should be included to achieve a more natural and stable biped stance simulation, which would enable more physiologically plausible muscle activations.

In addition, our current postural control model can only be used to simulate quiet standing; how it will deal with disturbances should also be investigated. Before that, however, a more sophisticated model that incorporates more physiologcally plausible components or mechanism should be created. Firstly, the fb controller should incorporate more fb information, such as visual and vestibular sensory inputs. Secondly, Mergner has noted that sensory inputs may have a threshold and be affected by noise [20], and a sensory integration and disturbance anticipatory mechanisms may exist. Such a mechanism should therefore be employed in our postural control model to investigate the ff input response to this mechanism as a means of maintaining balance. In addition, other important indicators for stance postural control such as the sway path, sway density, and power spectral density should be investigated in the future to evaluate the model.

Further, whether the model is overfitting or not should be validated. The NC model coordinates 35 muscles (left and right muscles are controlled symmetrically) to maintain the musculoskeletal model in a stance posture. However, only 8 of 35 simulated muscle activations were compared to experimental data. More muscle activation data should be measured in the experiment, and then be used to validate the NC model. Moreover, the complexity of the NC model and musculoskeletal model necessary to study bipedal stance postural control should also be investigated.

A final limitation is that the comparison between the simulated results and experimental data was inaccurate, owing to the difference in the weights, heights, and stance posture of the musculoskeletal model and the experimental subjects. The comparison was also affected by the EMG data normalization method employed by Panzer et al. [71]. In future, experiments on human quiet standing should be conducted as part of the controller design project, with the experimental setup matching the simulation conditions.

## Conclusion

An NC model was developed to generate a human-like biped stance. Rather than an inverted pendulum, a musculoskeletal model was used to approximate the human anatomy. The NC model utilized in the postural control model consisted of ff and fb controls. Further, a variable design framework was developed for the NC model so as to maintain the musculoskeletal model in a standing position under the influence of a 120-ms neurological time delay. The NC model generated physiologically plausible muscle activations for the biped stance. The NC model also reflected a salient physiological feature, i.e., that humans select a low active stiffness level during standing so as to achieve low energy consumption.

## Supporting Information

S1 Video Biped stance simulation video. (MP4)
